# Volume Pre-Allocation Strategy for Enhancing Formability and Die Life in AISI-410 Martensitic Stainless Steel U-Shaped Forgings

**DOI:** 10.3390/ma18163866

**Published:** 2025-08-18

**Authors:** Zhuo Deng, Biao Guo, Qifeng Tang, Zhangjian Zhou, Xinggui Wang, Jiupeng Song, Yu Zhang

**Affiliations:** 1 Key Laboratory of Materials and Surface Technology, Ministry of Education, School of Materials Science and Engineering, Xihua University, Chengdu 610039, Chinatqfkmust@126.com (Q.T.);; 2 School of Materials Science and Engineering, University of Science and Technology Beijing, Beijing 100083, China; 3 Sichuan Yuteng Machinery Forging Co., Ltd., Guangan 638500, China

**Keywords:** martensitic stainless steel, U-shaped forgings, Finite Element Analysis, volume pre-allocation, forging die wear

## Abstract

To address incomplete die filling, high cracking tendency, and severe die wear in the conventional forging of AISI-410 martensitic stainless steel U-shaped forgings, an optimized billet volume pre-allocation strategy was proposed. Two improved forging schemes for the U-shaped forgings were designed: the Arc Concave Flattening Scheme (adding arc-shaped concave features to the flattening die for corner volume compensation) and Preformed Volume Allocation Scheme (incorporating a preforming step for strategic volume pre-allocation at ends and corners). Finite Element Analysis employing the Oyane damage model and Archard wear model was employed to simulate and optimize the forging process. The optimal scheme was applied to production trials. The results demonstrated that the Preformed Volume Allocation Scheme significantly improved the geometric compatibility between the billets and the final forging die cavity. As a result, the billet’s temperature, strain, and equivalent stress uniformity increased, reducing cracking tendency. Moreover, the rise in the mitigated temperature and stress concentration resulted in reduced final forging die wear. Production trials confirmed a qualified rate of ~96% (34% higher than the Original Scheme). The final forging die service life reached 300 pieces per refurbishment cycle, showing a 50% improvement. This work provides theoretical and practical guidance for optimizing the forging processes of complex martensitic stainless steel components.

## 1. Introduction

The key components used in aerospace, navigation, petrochemical, and other fields are crucial for the safe and reliable operation and stable maintenance of their equipment. These key components must possess high strength, stability, and excellent corrosion resistance. Therefore, these components are typically manufactured from corrosion-resistant, high-strength martensitic stainless steel forgings [1,2,3,4]. However, these components not only consist of special materials but often feature complex geometries as well, making them difficult to forge. They are prone to low forging qualification rates and short service lives of forging dies [5,6,7,8]. Therefore, research on the forging process of complex martensitic stainless steel forgings for equipment in the aforementioned fields holds significant practical engineering value.

Martensitic stainless steels exhibit high deformation resistance and poor formability, especially when forging complex geometries. Consequently, challenges such as incomplete die filling, damage-induced cracking, and severe forging die wear are often encountered in actual forging, which significantly compromise the forming quality and stability of the forgings, reduce the life of the forging die, and escalate the production costs. In forging martensitic stainless steel engine valves, Ji et al. [9] employed a cross-wedge rolling plus forging process to replace the traditional extrusion plus forging and electric upsetting plus forging methods. Utilizing the high-speed rotation and precise temperature control characteristics of this process significantly improved the filling integrity of the valve forgings and effectively reduced the risk of incomplete die filling. Dourandish et al. [10] studied the die forging of martensitic stainless steel turbine shafts. They found that forgings tended to crack when forged at excessively high temperatures, while the cracking tendency decreased significantly at lower temperatures. Lisiecki et al. [11] further found that martensitic stainless steel exhibits good forgeability when forged at high strain rates within the warm forging range.

In addition, Kitayama [12] investigated the effect of the structure of the contact surface between the billet and the forging die on die wear, noting that the complex structure of the contact surface can induce local high stress and temperature, aggravating die wear. He emphasized that the forming quality and forging die life of complex forgings can be significantly improved by using multi-step forging dies with different structures to regulate the flow of billet material. Gronostajski et al. [13] further found that die wear during the forging of martensitic stainless steel gears is significantly influenced by the properties of the billet–die contact surface, including contact time, temperature, and stress distribution. These factors collectively determine the contact time and corresponding temperature changes at different locations on the die surface. A longer contact time results in higher die surface temperatures, thereby increasing susceptibility to wear. If high-stress areas occur, wear is further exacerbated.

Therefore, actual production requires careful consideration of multiple factors affecting both forging quality and die wear. Given this, adopting Finite Element Analysis (FEA) with multi-physical field coupling, to account for the combined effects of these factors, is an effective approach. This method optimizes forging processes and die designs, reduces trial-and-error costs, and improves production efficiency. Yin et al. [14] utilized FEA to optimize the multi-directional die forging sequence of copper alloy valve bodies, thereby improving workpiece material flow uniformity, preventing punch fracture, reducing die wear, and enhancing forging quality. Łukaszek-Sołek et al. [15] optimized the die forging scheme and die design for martensitic stainless steel gears via FEA, addressing problems like incomplete die filling, damage-induced cracking, and severe die wear in hot die forging, thereby improving gear quality and extending die service life.

**Figure 1:**
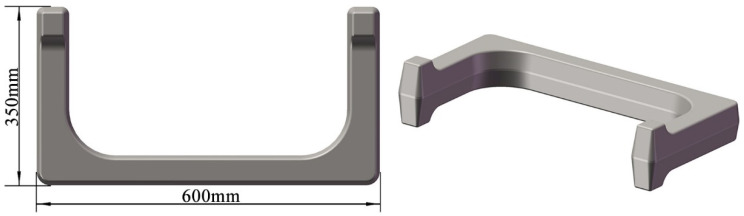
Model of U-shaped forging.

Although the above research provides important insights into improving the forging process of martensitic stainless steel complex forgings, the development of martensitic stainless steel forgings with a unique U shape still faces significant challenges. Figure 1 shows the model of martensitic stainless steel U-shaped forging. In the Original Scheme, the forging process used round steel for cutting, and then stainless steel is formed through the process steps of flattening, bending, and final die forging in sequence. This process is prone to the following problems:

1. The volumes of the two ends and the corners of the U-shaped forgings are significantly larger than those of other parts (Figure 1). However, the volumes of the billets at the corresponding ends in the Original Scheme do not increase (Figure 4(a1–a5)), and the volumes of the billets at the corresponding corners are reduced after bending (Figure 4(a1–a5)). Such unreasonable volume allocations of the billets can easily result in the appearance of too-narrow flashes around the ends and corners of the forgings (Figure 4(a1–a5)), incomplete die filling, and even large deformation and damage accumulation, reducing the forming quality of the forgings.
2. In the Original Scheme, the billets are simple round rods. During the die forging process after the press bending (Figure 4(a1–a5)), the billets undergo severe deformation, which easily results in high deformation heat and stress, as well as severe friction with the forging die surfaces, accelerating forging die wear and shortening the service life of the forging die. The practices of producing U-shaped forgings using the Original Scheme showed that the flashes around the forging ends and corners were too narrow, the ends were not prone to be filled completely, and the corners were prone to cracking, with severe die wear observed.

These problems encountered in the practical production of the U-shaped forgings are closely associated with forging schemes and die design. The literature review provides valuable insights and methodologies for optimizing the initial billet state and die structure to enhance the forming quality of forgings and reduce forging die wear. This work focuses on the practical problems encountered in producing AISI-410 martensitic stainless steel U-shaped forgings, such as a low forging qualification rate and a short die service life. By combining FEA with production trials, the hot deformation behaviors of the AISI-410 martensitic stainless steel under forging process conditions were systematically investigated, along with the influence mechanisms of temperature and stress–strain states of the billets in different multi-step forging schemes on forging forming quality and forging die wear. Based on this, an optimized forging scheme was proposed. Ultimately, production trials validated the correctness and effectiveness of the FEA results and the optimal forging scheme. It should be noted that this study was focused on macroscopic process optimization and industrial applicability, while microstructural characterization (e.g., EBSD analysis) and detailed production line configurations fall beyond the scope of the present work.

## 2. Materials and Methods

### 2.1. Materials and Modeling for Finite Element Analysis

In this work, the professional FEA software Deform-3D v10 for metal plastic forming was used to perform FEA on the forging process of the U-shaped forgings [16]. The forging material was AISI 410 martensitic stainless steel, and the principal mechanical properties and chemical composition are shown in Table 1 and Table 2. According to the deformation temperature and rate range of the workpiece in the forging process, the stainless steel was subjected to a Gleeble hot compression test at deformation temperatures of 1050 °C, 1100 °C, and 1150 °C and strain rates of 0.1 s^−1^, 1 s^−1^, and 10 s^−1^.

**Figure 2:**
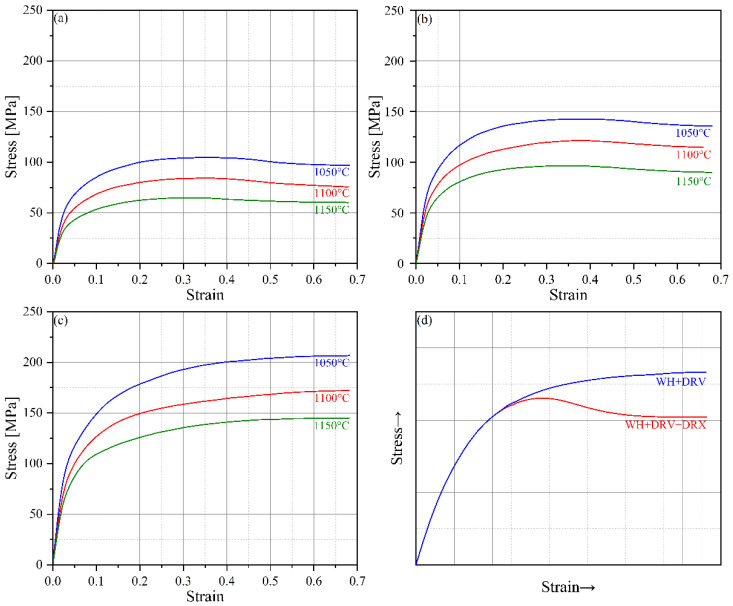
Hot compression stress–strain curves at different strain rates of (**a**) 0.1 s^−1^, (**b**) 1 s^−1^, and (**c**) 10 s^−1^ and (**d**) typical stress–strain curves of different deformation mechanisms.

The measured rheological behavior data (stress–strain curves) are shown in Figure 2. The flow stress of the stainless steel decreased with increasing deformation temperature and decreasing strain rate and increased conversely. This behavior was related to the effects of deformation temperature and strain rate on the work hardening (WH), dynamic recovery (DRV), and dynamic recrystallization (DRX) of the metal [17]. Higher deformation temperatures and lower strain rates can easily induce DRV and DRX in metals. The softening effect of DRV and DRX can effectively offset the influence of WH and enhance the softening ability of metals. Once the softening effect of DRV and DRX exceeds the hardening effect of WH, the flow stress will no longer increase but begin to decrease and reach a peak value. When the softening effect of DRV and DRX is balanced with the hardening effect of WH, the flow stress will be in a steady state (Figure 2a,b). Therefore, the high-temperature plastic deformation mechanism of the metal in this case mainly includes WH, DRV, and DRX, as shown in Figure 2d. However, the lower deformation temperature and higher strain rate mainly induce DRV in metal. The softening effect caused by DRV is not enough to completely offset the hardening effect of WH, resulting in the continuous increase in flow stress at a decreasing rate with increasing strain (Figure 2c). Therefore, in this case, the high-temperature plastic deformation mechanism of the metal mainly includes WH and DRV, as shown in Figure 2d. In general, AISI-410 martensitic stainless steel has three plastic deformation mechanisms of WH, DRV, and DRX at low strain rates (0.1 s^−1^ and 1 s^−1^) within the forging temperature range, and the plastic deformation mechanisms of WH and DRV mainly occur at high strain rates (10 s^−1^).

Based on the above analysis, the stress–strain data of AISI-410 martensitic stainless steel, measured by Gleeble hot compressions, were imported into the Deform-3D FEA software to analyze the flow and filling behaviors of the billets during the forging process of the U-shaped forgings. Moreover, the Oyane damage model (Equation (1)), considering the effect of stress triaxiality, was used in the FEA to predict the damage accumulation distribution of the billets during the forging process [18]. The Archard wear model (Equation (2)) was used to analyze the forging die wear distribution of the U-shaped forging [19]. The forging die material is AISI H-13 steel (Chengdu Huaxia Chemical Reagent Co., Ltd., Chengdu, China) (equivalent to DIN 1.2344 per DIN 17006:2021-08 (System for material designation, Deutsches Institut für Normung, Berlin, Germany), EN X40CrMoV5-1 per EN 10027-2 (Designation systems for steels, European Committee for Standardization, Brussels, Belgium), and GB/T 1299 4Cr5MoSiV1 per GB/T 1299-2014 (Alloy tool steels, Standardization Administration of China, Beijing, China)). In addition, the contact heat transfer between the billets and the forging dies and the heat dissipation of the billets in the air during the forging process have a significant influence on the temperature of both the billets and the forging dies. Accordingly, in the FEA, the heat transfer coefficient between the billets and the forging dies along with the heat dissipation coefficient of the billets in the air were determined based on empirical data and the research findings of Hartley et al. [20], Blau [21], and Jin et al. [22]. Table 3 shows the parameters used in the FEA of the forging process of the U-shaped forgings. In the meshing of the billets, the complex deformation areas are locally refined. At the same time, the adaptive meshing function of the FEA software is used to dynamically adjust the mesh of the billets during the deformation process to automatically compensate for the volume loss caused by the mesh redivision.

Model formula:

1. Oyane damage model

The damage accumulation *D* can be calculated by the following formula:

(1) $$D=\int 1+\alpha \frac{\sigma_{m}}{\sigma_{e}}{d\epsilon }_{p}$$

where

$\sigma_{e}$ is the equivalent stress (MPa);

${d\epsilon }_{p}$ is the cumulative plastic strain increment (dimensionless);

α is an empirical constant representing the sensitivity of damage accumulation, and its value is 3 (dimensionless) [27,28,29];

$\sigma_{m}$ is the hydrostatic pressure, and the calculation formula is$\;\sigma_{m}=\frac{\sigma_{1}+\sigma_{2}+\sigma_{3}}{3}$, where $\sigma_{1}$, $\sigma_{2}$, and $\sigma_{3}$ are the principal stress components (MPa);

When *D* ≥ 1, this indicates damage-induced cracking (dimensionless).

2. Archard wear model

The amount of wear *W* can be calculated by the following formula:

(2) $$W=\int K\frac{pv}{H}dt$$

where

**Figure 3:**
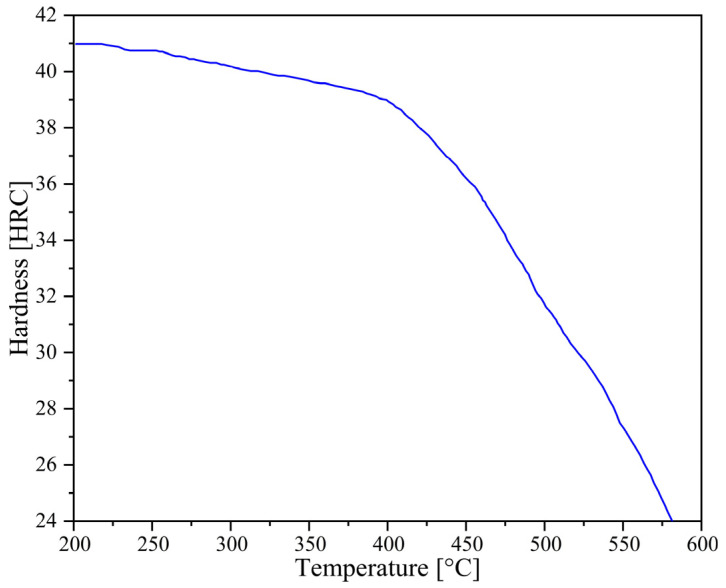
Hardness of H-13.

*p* is the interface pressure (MPa);
*v* is the sliding velocity (mm/s);
*t* is the contact time (s);
*K* is the wear coefficient, which is 1.73 × 10^−5^(HRC/MPa) [30,31];
*H* is the hardness of the forging die (HRC) (Figure 3) [32,33,34].

### 2.2. Forging Schemes of U-Shaped Forgings

**Figure 4:**
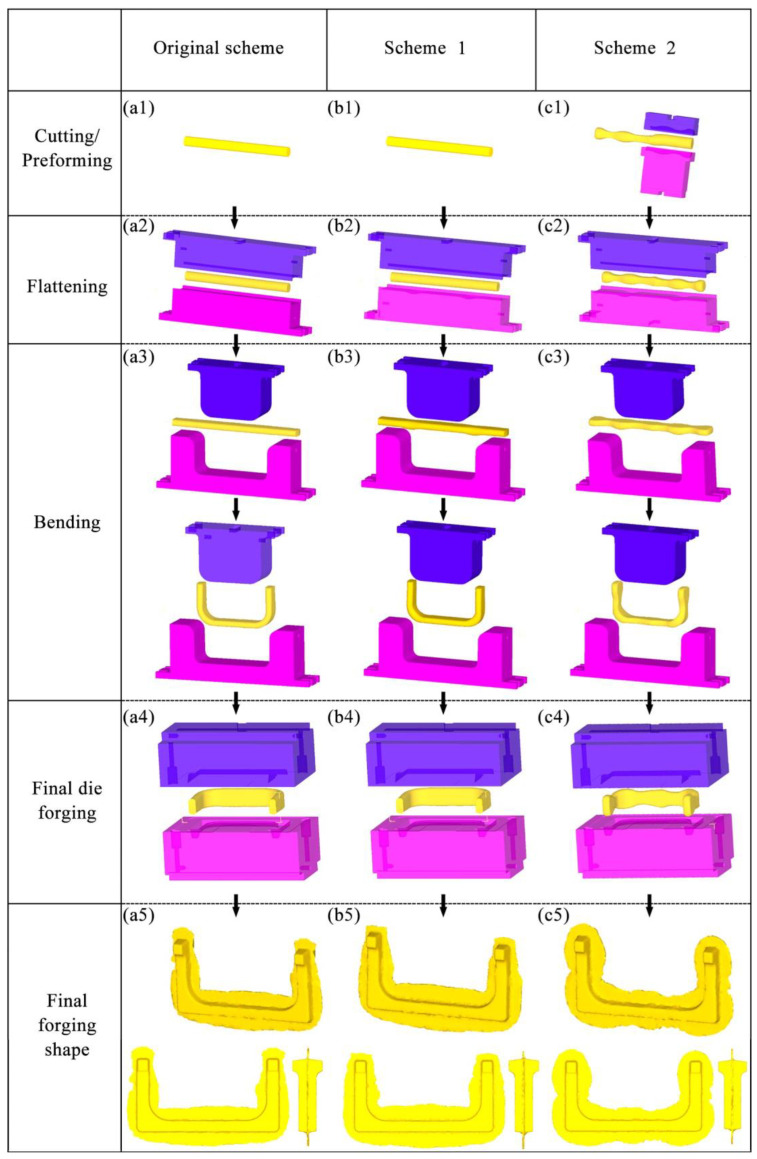
Forging schemes and final forged shapes of the U-shaped forgings: (**a1**–**a5**) Original Scheme; (**b1**–**b5**) Scheme 1; (**c1**–**c5**) Scheme 2.

Based on the analysis of the shape, size, and forming characteristics of the U-shaped forgings, three different multi-step forging schemes were designed. The initial billet was a cylindrical bar of a diameter of 10 mm and length of 72 mm. Figure 4(a1–a5) is the Original Scheme, which was served as a comparison for the subsequent improved Scheme 1 and Scheme 2. In the Original Scheme, the heated billet was first placed horizontally in a flattening forging die, and the flattening operation was performed radially. When the upper and lower flattening forging dies are closed during the flattening, the billet reaches the preset flattening amount, thus completing the flattening process step. Then, the billet is bent along the flattening direction using a bending forging die, causing the billet to gradually form a U shape, until the upper forging die stops moving, thereby completing the bending process step; finally, the U-shaped billet is placed horizontally in the forging die for final die forging. When the upper and lower forging dies are closed, the final die forging of the forging is completed. In the Original Scheme, the cross-sectional area of the corners of the billet is reduced from approximately 5757.3 mm^2^ to approximately 5178.3 mm^2^ during bending, and the volume is insufficient due to the bending operation. Therefore, it is easy to cause quality problems, such as too small flash at the corners of the forging and damage-induced cracking. Moreover, the volumes of the two ends of the target forging are larger than those of other parts of the forging, requiring more metal filling (Figure 1). However, in the Original Scheme, the amount of metal at the two ends of the billet is much smaller than that of the target forging, which may cause the two ends of the forging to be not fully filled, impairing the forming quality of the forging. The shape of the forging after the final die forging is shown in Figure 4(a1–a5).

**Figure 5:**
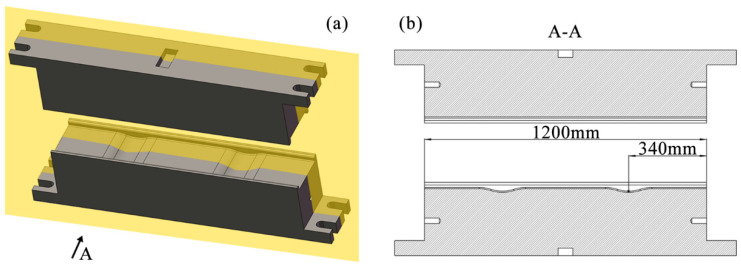
Flattening forging die in Scheme 1: (**a**) free view and (**b**) cross-sectional view.

In view of the possible forming quality problems at the corners of the forging in the Original Scheme, Scheme 1 (Arc Concave Flattening Scheme) was designed as shown in Figure 4(b1–b5). This scheme combines the shape and size of the corners of the target forging and adds two symmetrical arc-shaped concave features on the lower flattening forging die, as shown in Figure 5a. The positions of the two arc-shaped concave features correspond to the positions of the corners of the U-shaped forging, aiming to increase the volume of the billet at the corners so that the billet can form full corners and sufficient flashes during the final die forging, thereby improving the forming quality of the forging. Therefore, after the volume of the billet is pre-allocated in Scheme 1, it is expected that effective flashes can be formed at the corners of the forging. However, the two ends of the forging still face the risk of incomplete die filling, as shown in Figure 4(b1–b5).

**Figure 6:**
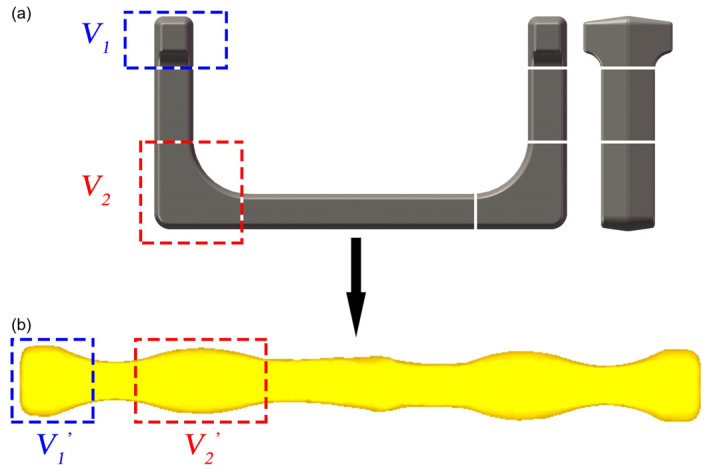
Preform design flow chart: (**a**) volume distribution diagram of the ends and corners of the U-shaped forging and (**b**) volume distribution diagram of the corresponding positions of the billet after free forging.

Based on the analysis of the Original Scheme and Scheme 1, Scheme 2 (Preformed Volume Allocation Scheme) is proposed to optimize billet volume pre-allocation through a dedicated preforming step. This scheme incorporates strategic volume pre-allocation at the ends and corners, as shown in Figure 4(c1–c5) and detailed in Figure 6. This scheme introduces a multi-directional forging operation before the flattening step of Scheme 1 to strategically form protruding features with intentionally allocated excess volumes (V_1_′ at ends and V_2_′ at corners) at critical billet locations. These preformed volumes (Figure 6b) are designed to precisely match the geometric requirements of high-fill-demand regions in the target U-shaped forging (Figure 6a), accounting for both the final forged geometry (V_1_, V_2_) and the necessary flash compensation volume. The temperature of the billet may drop due to its heat loss during this preforming process. Therefore, if necessary, the billet needs to be heated again to ensure that it has a suitable temperature in the subsequent forging process. Mesh sensitivity analysis was conducted to ensure the result stability. Three mesh densities (40,000, 60,000, and 80,000 elements) were tested for the final die forging step. The critical outputs (equivalent strain, stress, damage, and wear depth) varied by less than 5% between 60,000 and 80,000 elements (Table 4), confirming that the 60,000-element mesh provided sufficient accuracy while balancing computational efficiency. In this scheme, the added preforming step makes the volume allocation of the billet closer to that of the target forging, thereby reducing the risk of incomplete die filling and addressing problems such as excessively small flash. In addition, this scheme is expected to reduce the lateral flow of the billet in the final forging, reduce the risk of the damage-induced cracking in the forging, and reduce the wear of the final forging die.

## 3. Results and Discussion

### 3.1. Analysis of FEA Results

Before the production trials, the multi-step forging process of the U-shaped forgings in the three schemes was simulated by FEA. The temperature, stress, and strain distributions of the billets, as well as the damage accumulation of the final forgings and the wear tendency of the final forging die, were analyzed to obtain the optimal forging scheme for the U-shaped forging, improving the forging forming quality and extending the life of the forging die.

#### 3.1.1. Temperature Field Analysis

A reasonable billet temperature is an important factor in ensuring that the final forgings achieve good microstructure and mechanical properties. Additionally, a reasonable temperature and its uniform distribution can also enhance the plasticity of the metal, promote the flow and filling of the billets, and prevent defects such as folding and cracking during the forging process [35].

**Figure 7:**
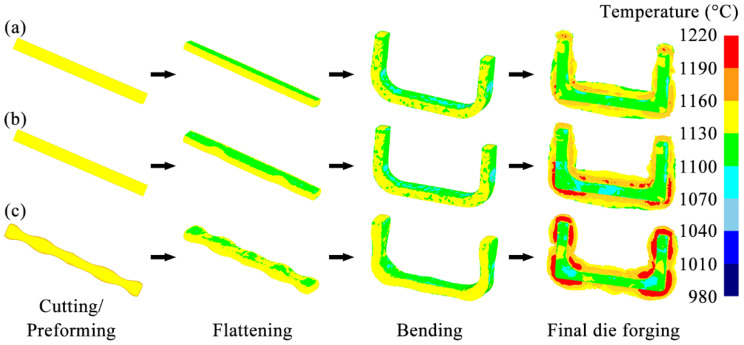
Temperature distributions of the billets during multi-step forging: (**a**) Original Scheme; (**b**) Scheme 1; (**c**) Scheme 2.

Figure 7 shows the temperature distributions of the billets at each forging step in three schemes. In each scheme, the temperature distribution of the forging body was relatively uniform, roughly maintained within the required range of 1050–1150 °C. After the billet underwent the flattening and bending steps, the areas with lower temperatures were mainly concentrated on the surface in contact with the forging die. This lower temperature is due to the heat transfer between the billet and the forging die. After the final die forging step, the flash was severely deformed and generated much heat, so the temperature of the flash of the billet was higher than that of other areas.

The temperatures of the two ends of the billets in the Original Scheme and Scheme 1 are relatively high (note: Scheme 1 = Arc Concave Flattening Scheme; Scheme 2 = Preformed Volume Allocation Scheme). These high temperatures appear because the volumes of the ends of the billets in the Original Scheme and Scheme 1 are small. Accordingly, the contact time between the ends and the inner surface of the forging die cavity during the final forging step is short, and the contact surface is small, resulting in less heat transfer between the ends and the forging die, so the temperature is relatively high. Similarly, after the billet in Scheme 2 has undergone the final die forging step, a relatively high-temperature area also appeared in the middle of the two arms of the forging. However, in Scheme 2, the temperature distributions of the billet at the two ends and the corners were more uniform. High-temperature flashes, formed by severe plastic deformation, appeared around both locations. This temperature distribution characteristic indicates that the plastic deformation of the billet at the ends and corners is more uniform, and the die filling is more complete. This state helps to reduce the risks of defects, such as uneven microstructure and even cracking at these two locations of the forging, thereby achieving good forming quality.

#### 3.1.2. Equivalent Strain Analysis

During the forging process, the strain distribution gradient affects the uniformity of the microstructure, residual stress, filling integrity, and crack resistance of the forging. So, the strain distribution gradient is a key indicator for evaluating the uniform deformation ability of the forging. The increase in the strain distribution gradient denotes the aggravated heterogeneity of the local deformation, which will reduce the overall forming quality of the forging [36].

**Figure 8:**
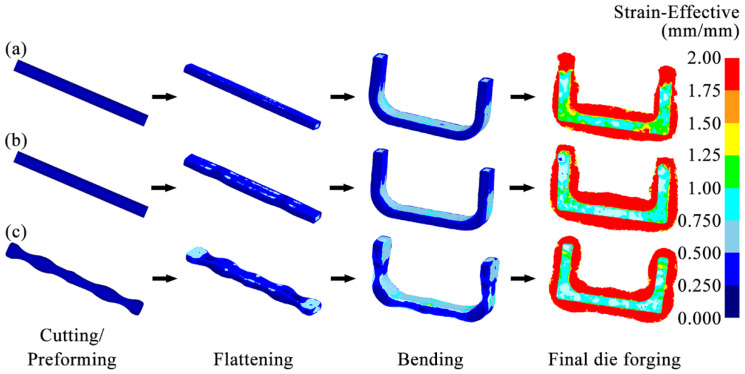
Equivalent strain distributions of billets during multi-step forging: (**a**) Original Scheme; (**b**) Scheme 1; (**c**) Scheme 2.

Figure 8 shows the equivalent strain distributions of the billets at each forging step in three schemes. After the billet was subjected to the flattening and bending steps, its relatively high strain appeared in the area close to the forging die in each scheme. However, after the billet was subjected to the final die forging, its strain distribution in each scheme was not the same. In the Original Scheme, a high strain area greater than 1.75 appeared near the corners of the forging body, indicating an excessively large strain gradient. However, the flash near the corners was abnormally narrow. This high strain and narrow flash distribution indicated that the billet in the Original Scheme had a poor match with the forging die cavity at the corners, which neither ensured that the forging body at the corners was fully filled nor improved the local large strain gradient well, easily resulting in an uneven microstructure, incomplete die filling, and even damage-induced cracking and other defects. Scheme 1 used a flattening forging die with arc-shaped concave features to increase the volume of the billet at the corners so that a larger flash was formed near the corners, and the strain gradient of the forging body at the corners was effectively improved, helping to improve the forming quality of the forging corners. However, the utilization of the flattening forging die with two arc-shaped concave features not only directly increased the volume of the forging corners but indirectly increased the volume of the horizontal section of the forging as well, resulting in the formation of a wider flash in the horizontal section of the billet after final die forging.

Moreover, both the Original Scheme and Scheme 1 exhibited excessive strain gradients and insufficient flashes at the end regions of the billet. This phenomenon indicated poor geometric compatibility between the billet ends and the die cavity in the two schemes, thereby predisposing the forging ends to microstructural inhomogeneity, incomplete die filling, and even damage-induced cracking. Furthermore, unlike the Original Scheme, Scheme 1 employed a flattening die with arc-shaped concave features. This die structure caused the greater volume of the billet towards the corners and horizontal sections, resulting in reduced end volume compared with the Original Scheme. As a result, the excessively high and low strain appeared at the billet ends after the final die forging. Such a steep strain gradient demonstrated that while Scheme 1 improved the formability of the billet corners, it further compromised geometric compatibility at the billet ends with the final forging die relative to the Original Scheme, ultimately degrading the overall formability quality of the forging.

On the contrary, Scheme 2 fully pre-allocated the volume of the billet through the preforming step, increasing the volume of the billet at the corners and the ends to improve the matching degree between the billet and the forging die cavity. This more reasonable volume allocation enabled the billet to form a sufficiently broad and uniform contour-adaptive flash around the forging body. Moreover, the strain distribution of the forging body was significantly improved, reduced to 0.75–1.5. The overall filling of the forging was complete. This uniform strain distribution and good filling effect helped to improve the forming quality of the entire forging.

#### 3.1.3. Equivalent Stress Analysis

The billet is subjected to complex multi-directional stresses during the forging process. Equivalent stress can reflect the comprehensive effect of complex stresses on the billet. Therefore, equivalent stress was an important basis for analyzing the stress conditions of the billet and problems related to the forging process [37].

**Figure 9:**
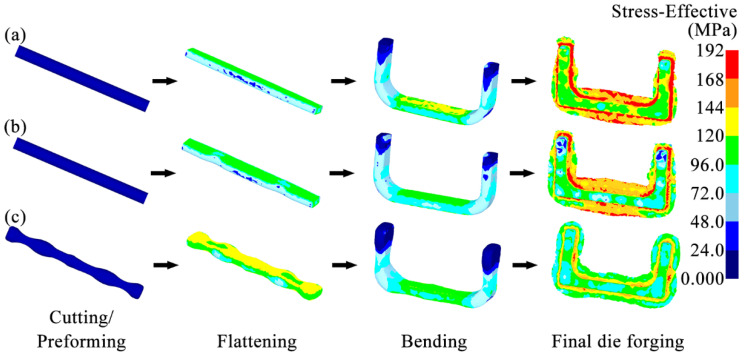
Equivalent stress distributions of billets during multi-step forging: (**a**) Original Scheme; (**b**) Scheme 1; (**c**) Scheme 2.

Figure 9 shows the equivalent stress distributions of the billets at each forging step in the three schemes. After the billet was subjected to the flattening and bending steps, its stress was mainly concentrated in the part directly contacting the forging die in each scheme. In the Original Scheme, after the billet was subjected to the final die forging, the high-stress area was mainly concentrated in the flashes and the areas where the flashes were combined with the forging body. However, the stress distribution in the forging body was uneven, with obvious stress concentrations in several areas. As a result, a stress peak of approximately 190 MPa was observed. This highly concentrated stress can easily cause forging damage and wear on the forging die.

Scheme 1 utilized a flattening forging die with arc-shaped concave features to pre-allocate the volume of the billet, thereby ensuring a more matched shape and size between the billet and the forging die cavity. Although the high stress of the billet in this scheme was also concentrated in the flashes and the areas where the flashes were combined with the forging body, the stress peak of the forging body was significantly reduced to about 143 MPa. This improvement in the stress concentration helps to suppress the forging damage and forging die wear. However, the stress value of the billet at the two ends was too low, at only about 38 MPa. This too-low stress indicated that the billet was not deformed sufficiently during the forging process or even not completely filled (corresponding to the lower strain at the two ends in Figure 8b), which will seriously affect the forming quality of the forging at this position.

In Scheme 2, the high stress of the billet was mainly distributed at the junction of the forging body and the flashes. The scheme adopted the preforming step to more reasonably pre-allocate the volume of the billet. The reasonable volume allocation of the billet significantly improved the stress distribution of the final forging body, making the stress distribution more uniform and the stress peak value only about 112 MPa. That is, the peak stress of the forging body was reduced by 41%. Therefore, Scheme 2 can ensure complete filling of the forging and suppress forging damage and forging die wear.

#### 3.1.4. Forging Damage Analysis

Damage is used to assess the cracking tendency of the billet during forging. A larger damage value means a higher risk of cracking. Forgings formed by the multi-step forging often undergo complex plastic deformation, and their damage will gradually accumulate [38]. Therefore, this work was focused on the damage accumulation of U-shaped forgings after final die forging.

**Figure 10:**
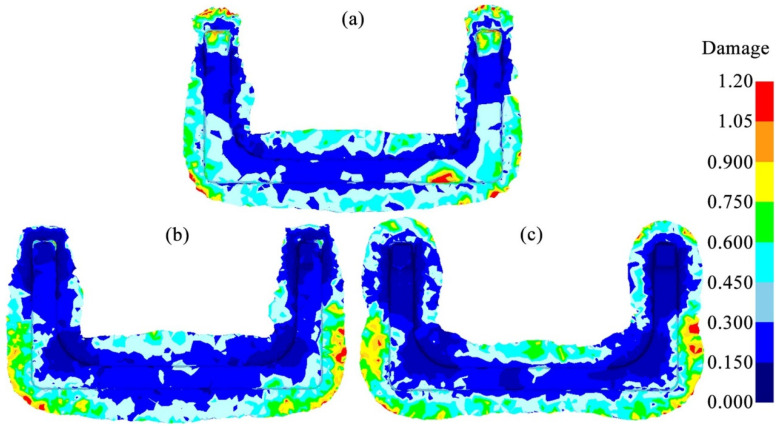
Comparison of damage in U-shaped forgings: (**a**) Original Scheme; (**b**) Scheme 1; (**c**) Scheme 2.

Figure 10 shows the damage distributions of the billets after the final die forging in the three schemes. In the Original Scheme, there were areas with damage values greater than 1.0 around the ends and corners of the billet, suggesting that the billet had a high tendency to crack. These high-damage areas overlapped with the high-strain areas in Figure 8. This phenomenon was consistent with the law of damage accumulation due to deformation. It was also highly consistent with the Oyane damage model, which states that the damage value *D* is proportional to the equivalent plastic strain increment dϵ_p_. The high plastic strain generated during the forging process of the billet often causes the exponential growth of dislocations. These dislocations would accumulate at grain boundaries and second-phase particles, such as carbides, causing local stress concentrations that induce the nucleation of voids and form crack sources, ultimately resulting in the cracking of the billet.

Scheme 1 pre-allocated the volume of the billet based on the Original Scheme to improve the deformation uniformity of the billet (Figure 8) while ensuring that there was sufficient metal filling at the corners of the forging, thereby reducing forging damage. However, although the damage at the corners was significantly reduced compared with the Original Scheme, there was still a region with a higher damage value of 0.6 to 0.75 in the main body of the forging. Therefore, the cracking tendency was relatively high. In addition, although the damage at the ends of the forging was significantly improved in Scheme 1, this was mainly due to the low plastic strain at the ends (Figure 8), which in turn posed the risk of incomplete die filling.

On the contrary, Scheme 2 utilized the preforming step to further pre-distribute the volume of the billet at the corners and ends of the forging, which significantly improved the adaptability of the billet to the forging die cavity. As a result, a sufficiently broad contour-adaptive flash was formed near the ends and corners where damage-induced cracking was likely to occur. The entire flash edge was neat, providing good protection for the forging. This result made the damage distribution of the forging body more uniform. The overall damage value was less than 0.6, with a lower damage-induced cracking tendency, helping to improve the forming quality of the forging.

#### 3.1.5. Final Forging Die Wear Analysis

Wear is the primary failure mechanism of hot forging dies, accounting for approximately 70% of forging die failures. These wear failures can be attributed to high temperatures, high pressures, and complex loads on the working surface of the forging die during the forging process [39]. The actual application of the Original Scheme of the U-shaped forging showed that the wear failure of the final forging die mainly occurred in the flash groove bridge part of the lower forging die. This result was due to the flash groove bridge part having a special structure that was subjected to the coupling effects of various states of stress, temperature, and friction. Thus, its service condition was extremely bad. Accordingly, the wear tendency of the flash groove bridge part of the lower forging die of the U-shaped forging in three schemes was studied in detail to improve the service life of the final forging die in this work.

**Figure 11:**
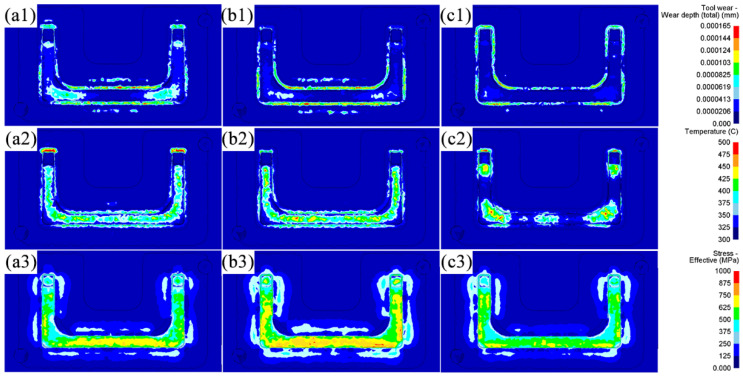
Comparison of wear, temperature, and stress of the final lower forging die in U-shaped forgings: (**a1**–**a3**) Original Scheme; (**b1**–**b3**) Scheme 1; (**c1**–**c3**) Scheme 2.

Figure 11 shows the temperature, stress, and wear distributions of the lower forging die after the final die forging step in each scheme. The severe wear area with a wear depth exceeding 0.000103 mm after one forging in the three schemes mainly appeared in the flash groove bridge part (Figure 11(a1,b1,c1)). There are three main reasons for this result: Firstly, the billet underwent severe plastic deformation in the flash groove bridge part, generating deformation heat (Section 3.1.1), which caused the temperature of the flash groove bridge part to rise (Figure 11(a2,b2,c2)). In particular, in Scheme 1, the billet formed a large area of flash in the middle horizontal section (Section 3.1.2), resulting in the highest temperature of the flash groove bridge part in the horizontal section of the final forging die. A high temperature reduced the hardness of the forging die and was more likely to aggravate forging die wear. Secondly, when the billet overflowed the die cavity, its flow was blocked at the flash groove bridge, which formed the high stress on the flash groove (Figure 11(a3,b3,c3)). In particular, in Scheme 1, the formation of a large area of flash (Section 3.1.2) caused the flash groove bridge to be subjected to high stress in several places (Section 3.1.3). Thirdly, the billet passed through the flash groove bridge at a high velocity, accompanied by high temperature and high stress (Section 3.1.1 and Section 3.1.3), causing rapid and intense friction with the flash groove bridge. Therefore, as the flash was formed and expanded during forging, the flash groove bridge was subjected to rapid and intense friction under high stress and high temperature. As a result, the wear of the flash groove bridge was continuously increased. These effects on the flash groove bridge corresponded to the Archard wear model.

In the Original Scheme and Scheme 1, the billets were less deformed at the ends, so the corresponding flash was smaller. Therefore, the wear of the final forging die in these two schemes was mainly concentrated at the corners and the flash groove bridge of the horizontal section. In particular, in Scheme 1, after the flattening forging flattened the billet die with the arc-shaped concave features, the volume of the horizontal section increased except for the corners, and a larger flash was formed in the horizontal section than in the Original Scheme (Figure 8). The formation of large-area flash induced high deformation heat, causing the flash groove bridge of the horizontal section of the forging die to heat up significantly (Figure 11(a2–c2)). The high temperature reduced the hardness of the forging die, which made it easier to aggravate the forging die wear. In addition, the formation of a large-area flash in the horizontal section of the billet in Scheme 1 caused the flash groove bridge to be subjected to high stress in several places (Figure 11(a3–c3)). Finally, under the actions of high stress, high temperature, and rapid and intense friction, the flash groove bridge in Scheme 1 was more severely worn than the Original Scheme (Figure 11(b1)). Friction sensitivity was evaluated by varying the friction factor (0.2–0.4): wear depth changed by 8% while maintaining the same trend across schemes. However, the wear of the flash groove bridge of the forging die in Scheme 2 was significantly reduced (Figure 11(c1)). This reduced wear was mainly due to the more reasonable pre-allocation of the billet volume by the preforming step so that each part of the billet was highly matched with the forging die cavity. It not only ensured the complete die filling of the forging but also formed a contour-adaptive flash with moderate width (Figure 8), reducing the stress, temperature, and friction of the flash groove bridge. As a result, the tendency of the forging die to wear was significantly reduced. Simultaneously, the wear depth prediction is supported by thermomechanical coupling: in Scheme 2, temperatures in 90% of the die area remain at 400 °C (Figure 7c), maintaining H13 steel hardness at HRC39 (Figure 3). This represents a significant improvement over the Original Scheme and aligns with the hardness–wear relationship in Archard’s model (Equation (2)).

#### 3.1.6. Forging Load and Impact Energy Analysis

Forming load and impact energy are important indicators for evaluating the economy of the forging process and the service life of the forging die [40,41,42]. This section presents a combination of the analyzed results of the temperature, strain, stress, and damage of the abovementioned billet with the forging die wear to explore the differences in forming load and impact energy among the three schemes. Accordingly, the mechanism of optimizing the forging scheme to reduce energy consumption and extend the die life was revealed.

**Figure 12:**
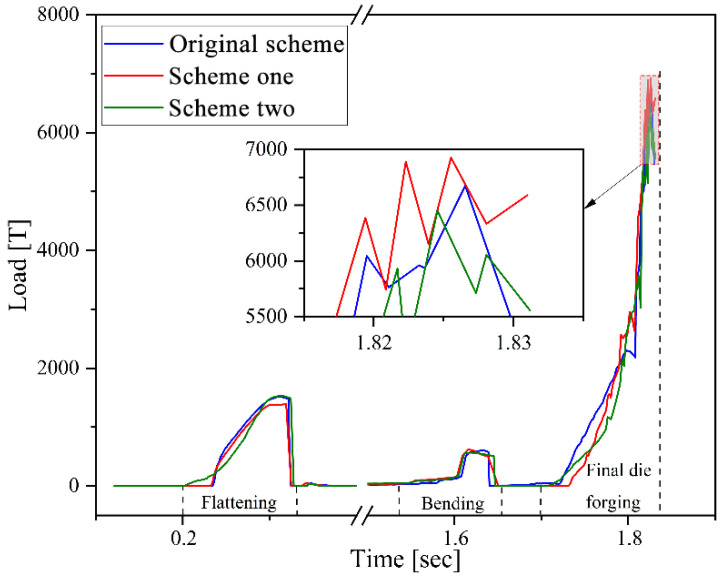
Forging load–time curves in the three schemes.

Figure 12 shows that the forging load–time curves of the U-shaped forgings in the three schemes have similar variation patterns. The load of the bending step was the lowest, followed by the flattening step, and the final die forging step reached the peak. Specifically, the maximum forging loads of the Original Scheme, Scheme 1, and Scheme 2 in the final die forging step reached approximately 6677 T, 6727 T, and 6453 T, respectively. Therefore, although Scheme 1 pre-allocated the billet volume based on the Original Scheme to improve the forming quality of the forging corners, a broad and uneven flash also formed around the corners and the adjacent horizontal section (Figure 8). This broad and uneven flash resulted in the load in the final die forging step of this scheme being the highest among the three. Scheme 2 adopted an optimized volume pre-allocation strategy so that the billet volume was more matched with the die cavity of the final forging die, which not only ensured that the forging was fully filled but also formed a uniform and reasonable flash, effectively reducing the load of the final die forging step. The reduction in load helped reduce the wear on the forging die.

During the multi-step forging of U-shaped forgings, the final die forging step induces the most severe plastic deformation in the billet, consequently requiring the highest energy input. Therefore, this work mainly examined the impact energy consumed at the final die forging step. The FEA results showed that the impact energies at final die forging in the Original Scheme, Scheme 1, and Scheme 2 were approximately 866 kJ, 766 kJ, and 766 kJ, respectively. This result showed that Scheme 1 and Scheme 2 employed the volume pre-distribution strategy to optimize the billet, which reduced the plastic deformation of the billet at the die forging step, thus effectively reducing the impact energy. In addition, although the FEA results showed that the impact energies at the final die forging steps of Scheme 1 and Scheme 2 were the same, Scheme 2 further pre-distributed the billet volume, improving the flow uniformity of the billet while reducing the peak forging load during the die forging process (Figure 12). Therefore, Scheme 2 not only created the best forging quality but also the least forging die wear among the three Schemes. Therefore, based on the comprehensive evaluation of forging damage, forging die wear, and forming load, Scheme 2 performed best in terms of improving the forging quality, extending the service life of the forging die, and reducing forging energy consumption, and it could be used for further production trials. Simultaneously, the load predictions align with the energy principles: the 766 kJ impact energy in Scheme 2 corresponds to a 6453 T load (Figure 12), representing a 3.4% reduction versus the Original Scheme.

#### 3.1.7. Mesh Sensitivity Validation

To verify the independence result from the mesh density, three discretization levels (40,000 to 80,000 elements) were compared for the final die forging step. As shown in Table 4, key parameters (strain, stress, damage, and wear) exhibited minimal fluctuations (<5%) when the mesh density increased from 60,000 to 80,000 elements. This confirms that the baseline 60,000-element model achieves stable predictions while maintaining computational practicality. All subsequent analyses used this validated mesh configuration.

### 3.2. Production Trials

**Figure 13:**
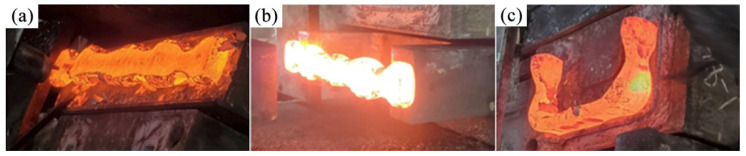
Actual forging process of U-shaped forging based on Scheme 2: (**a**) flattening; (**b**) bending; (**c**) final die forging.

**Figure 14:**
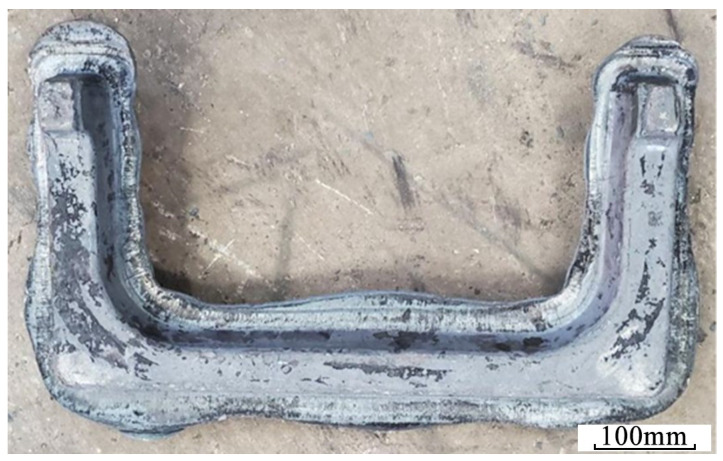
Photograph of U-shaped final forging.

To verify the effect of FEA, the production trials of U-shaped forgings were carried out using Scheme 2. First, the AISI-410 martensitic stainless steel bar was heated to the initial forging temperature of 1150 °C to form a pre-designed billet with protruding features. Subsequently, to compensate for the heat loss of the billet during the preforming process, it was reheated to 1150 °C before subsequent flattening. Finally, the billet that was heated second was subjected to the steps of flattening, bending, and final die forging using a 6300-ton screw press (Figure 13). Figure 14 shows the U-shaped final forging of Scheme 2. The main body morphology and flash distribution of the final forging were highly consistent with the FEA results in Section 3. Production trials evaluated forging quality through visual inspection for geometric completeness (full die filling), uniform flash distribution, and absence of defects (cracking/folding), showing that Scheme 2 significantly improved the forming quality. The trials involved 305 forgings per scheme, as statistically detailed in Table 5. Although the preforming step adds one heating cycle and ~15% processing time per piece, the 50% longer die life and 24.3% higher yield (Table 5) substantially offset this cost in mass production. The close agreement between predicted damage/wear (FEA) and actual defect-free forgings (trials) further validates the robustness of the Oyane/Archard models to parameter uncertainties. Similarly to the multi-stage bending process for large-scale coupler yokes proposed by Je et al. [9], the volume pre-allocation strategy (Scheme 2) addresses incomplete die filling and cracking by optimizing geometric compatibility between the billet and die cavity. However, while Je et al. [9] utilized rollers and slanting dies to reduce friction and strain concentration, our approach strategically pre-allocates material volume at ends/corners via preforming, achieving a higher forging qualification rate (96% vs. unreported in [9]) and extending die service life by 50% per refurbishment cycle. Furthermore, energy parameter sensitivity was confirmed by production trials: actual energy consumption (765 ± 42 kJ) matched the FEA predictions within 5.5% error.

Based on the above FEA and production trial results, the existing production line was optimized and adjusted according to Scheme 2 to complete the mass production of U-shaped forgings.

The qualified rates of the U-shaped forgings and the refurbishment cycle of their final forging die in the Original Scheme and Scheme 2 were separately counted and compared, as shown in Table 5. The statistical results showed that the qualified rate of U-shaped forgings produced using Scheme 2 was 34% higher than that of the Original Scheme. This highly aligns with the FEA-predicted significant reduction in damage values (D < 0.6 in Figure 10c) and improved stress distribution uniformity (41% reduction in peak stress in Figure 9c). The service life of the final forging die used in Scheme 2 was about 300 pieces/refurbishment cycle, which was significantly increased by 50% compared with about 200 pieces/refurbishment cycle of the Original Scheme, directly corresponding to the simulated trend of reduced wear depth in Figure 11(c1–c3).

Overall, it is feasible to adopt Scheme 2 for the mass production of U-shaped forgings. The FEA of the multi-step forging process of the U-shaped forging can guide the optimization of its actual process, effectively avoid the waste of time and resources caused by the trial-and-error method, significantly improve the forming quality of the forging, extend the service life of the forging die, help to improve the stability of the forging, and reduce production costs.

## 4. Conclusions

A billet volume pre-allocation strategy was developed in this work to address the problems of incomplete die filling, high cracking risk, and severe forging die wear in the traditional forging process of AISI-410 martensitic stainless steel U-shaped forgings. By combining FEA with production trials, a comparative analysis was conducted of the Original Scheme, Scheme 1 (Arc Concave Flattening Scheme—adding arc-shaped concave features to the flattening die), and Scheme 2 (Preformed Volume Allocation Scheme—incorporating a preforming step for strategic volume pre-allocation). The forming effects after multi-step forging and die wear were determined. The main conclusions are as follows:

1. The billet volume pre-allocation strategy significantly improved the forming uniformity of the U-shaped forgings. Scheme 2 achieved precise billet volume pre-allocation through a preforming step, significantly improving the compatibility between the billet geometry and the final forging die cavity compared with the Original Scheme. This optimized volume pre-allocation improved the temperature field uniformity of the billets during the final die forging, reduced the equivalent strain to 0.75–1.5, decreased the mean equivalent stress to about 112 MPa, and reduced the peak stress of the forging body by about 41%, significantly improving the forming uniformity of the forgings.
2. An optimized billet volume pre-allocation strategy was implemented in Scheme 2 to create a uniform contour-adaptive flash around the U-shaped forging body. As a result, the temperature, stress gradients, and mechanical load on the final forging die contact surfaces decreased, reducing the wear of the flash groove bridge of the final forging die compared with the Original Scheme. Production trials confirmed that the service life of the final forging die in Scheme 2 had reached about 300 pieces per refurbishment cycle, approximately 50% higher than that of the Original Scheme.
3. The optimized billet volume pre-allocation strategy was applied in Scheme 2 to reduce the damage value of the U-shaped forging body to below 0.6, significantly lowering the cracking risk of the forgings. Compared with the Original Scheme, the peak forging load in Scheme 2 was reduced by about 3.4%, and the impact energy consumption decreased by about 11.5%. Energy efficiency optimization was achieved while ensuring forging quality. Production trials confirmed that utilizing the optimized Scheme 2 produced U-shaped forgings with uniform contour-adaptive flash and no defects such as folding or cracking. The qualified rate of the forgings reached approximately 96% (based on 305 trials), which is about 24.3% higher than the Original Scheme. The qualified rate of the forgings reached approximately 96%, which is about 34% higher than the Original Scheme. These promising results aligned with the FEA predictions of improved strain field uniformity, stress concentration relief, and damage tendency suppression, verifying the reliability of multi-physics field coupling analysis and achieving significant synergistic optimization of the forging process and forming quality of the forgings.

The optimization framework proposed in this work for the multi-step forging process provides theoretical guidance for the high-quality forming of complex martensitic stainless steel forgings. Future research could further explore the influence of forging schemes on microstructural evolution (e.g., recrystallization) to achieve precise control of the process parameters and mechanical properties.

## Figures and Tables

**Table 1:** Principal mechanical properties of AISI-410 martensitic stainless steel.

Yield Strength (MPa)	Tensile Strength (MPa)	Elongation (%)	Hardness (HV)
260	470	30	145

**Table 2:** Chemical composition of AISI-410 martensitic stainless steel (wt%).

Fe	C	Si	Mn	P	S	Cr	Ni
Bal.	0.09	0.48	0.52	0.02	0.005	12.23	0.32

**Table 3:** Parameters used in the FEA [23,24,25,26].

Physical Parameters/Properties	Assigned Values/Setting
Initial billet temperature (°C)	1150
Initial die temperature (°C)	350
Environment temperature (°C)	20
Number of billet elements	60,000
Convection coefficient (W/m^2^·°C)	20
Heat transfer coefficient (W/m^2^·°C)	11,000
Friction factor between the billet and a die	0.3
Single blow energy of air hammer (N-mm)	5.5 × 10^7^
Single blow energy of screw press (N-mm)	5.4 × 10^8^

**Table 4:** Mesh sensitivity analysis results (final die forging, Scheme 2).

**Parameters**	**40,000 Elements**	**60,000 Elements**	**80,000 Elements**	**Variation (%)**
Max equivalent strain	1.92	1.75	1.73	1.1
Max equivalent stress (MPa)	218	190	185	2.6
Damage value (corner)	0.78	0.68	0.66	2.9
Wear depth (mm)	0.000121	0.000103	0.000099	3.9

**Table 5:** U-shaped forging qualified rate.

Scheme	Texture	Billeting Plan/Piece	Practical Production/Piece	Repairs/Piece	Qualified Rate/%
Original Scheme	AISI-410	305	305	86	71.8
Scheme 2	AISI-410	305	305	12	96.1

Definitions: Billeting plan: initial billet count prepared for forging. Practical production: actual forged pieces completed. Repairs: forgings requiring rework due to defects (incomplete filling, cracking, etc.). Qualified rate: [(Practical production − Repairs)/Practical production] × 100%. Refurbishment cycle: service life interval for die maintenance.

## Data Availability

The original contributions presented in this study are included in the article. Further inquiries can be directed to the corresponding author.
